# Kinetic and kinematic parameters associated with late braking force and effects on gait performance of stroke patients

**DOI:** 10.1038/s41598-023-34904-3

**Published:** 2023-05-12

**Authors:** Mizuho Ohta, Saori Tanabe, Junji Katsuhira, Makoto Tamari

**Affiliations:** 1 Department of Physical Therapy, Faculty of Rehabilitation, Reiwa Health Science University, Fukuoka, Japan; 2 Graduate Department of Human Environment Design, Faculty of Human Life Design, Toyo University, Tokyo, Japan; 3 Department of Rehabilitation, Seiai Rehabilitation Hospital, Fukuoka, Japan

**Keywords:** Biophysics, Medical research

## Abstract

Late braking force (LBF) is often observed in the late stance phase of the paretic lower limb of stroke patients. Nevertheless, the effects and association of LBF remain unclear. We examined the kinetic and kinematic parameters associated with LBF and its effect on walking. Herein, 157 stroke patients were enrolled. Participants walked at a comfortable speed selected by them, and their movements were measured using a 3D motion analysis system. The effect of LBF was analyzed as a linear relationship with spatiotemporal parameters. Multiple linear regression analyses were performed with LBF as the dependent variable and kinetic and kinematic parameters as independent variables. LBF was observed in 110 patients. LBF was associated with decreased knee joint flexion angles during the pre-swing and swing phases. In the multivariate analysis, trailing limb angle, cooperativity between the paretic shank and foot, and cooperativity between the paretic and non-paretic thighs were related to LBF (*p* < 0.01; adjusted R^2^ = 0.64). LBF in the late stance phase of the paretic lower limb reduced gait performance in the pre-swing and swing phases. LBF was associated with trailing limb angle in the late stance, coordination between the paretic shank and foot in the pre-swing phase, and coordination between both thighs.

## Introduction

Stroke causes long-term disabilities and limits various activities of daily living^1^. Among these, improvement in gait disturbance is reported to be a goal for many stroke patients with hemiplegia^2–4^. It has been reported that the walking speed of stroke patients is related to their life space^5^ and improved walking speed enhances community participation and quality of life^6,7^. Therefore, increasing walking speed is essential for the rehabilitation of stroke patients; thus, factors associated with walking speed, including the propulsion force (PF) and the anterior component of the ground reaction force (GRF) appearing in the late stance phase, are important for rehabilitation^8,9^. In recent studies on hemiparetic gait, the PF has been adopted as the primary outcome and attracted attention as a factor for improving walking speed^10,11^.

In stroke patients, the posterior component of the GRF may be observed just before the foot-off. The posterior component of the GRF occurring in the late stance phase is defined as the late braking force (LBF) relative to the first braking force (FBF) occurring in the early stance phase. LBF has been reported to be involved in the knee joint flexion angle reduction during the swing phase^12^, and may reduce gait performance. However, the causes of LBF and its effects on gait performance have not been thoroughly examined. It has been reported that PF in the late stance phase is related to the knee joint flexion angle in the pre-swing and swing phases^13,14^. Therefore, PF must be considered when examining the impact of LBF on gait performance.

According to a previous report, stroke patients with LBF had decreased PF during walking^15^. Therefore, the kinetic and kinematic parameters related to PF may also be factors influencing LBF. Factors associated with PF include ankle plantar flexion moment in the late stance phase^16,17^, anterior displacements of the center of pressure (COP) in the stance phase^18,19^, and trailing limb angle (TLA)^20–22^, which may also be related to the factors that cause LBF.

Previous studies have reported that in the late stance phase, the net impulse (the sum of propulsive and late braking impulses) is correlated with unusual leg flexor activity, suggesting a deficit in limb coordination^15^. Furthermore, it has been reported that LBF is more likely to occur in patients who are unable to shift their weight rapidly from the paretic limb^12^. Therefore, analyzing the coordination between bilateral lower extremities is important.

Continuous relative phase (CRP) has been widely used as an indicator of limb coordination between bilateral lower extremities during walking^23,24^. The root mean square (RMS) of CRP (CRP-RMS) in a specific phase during walking is a useful indicator of limb coordination between segments^25^. Therefore, CRP-RMSs of the paretic and non-paretic lower limbs in the pre-swing phase are necessary factors in investigating the kinetic and kinematic associations of LBF.

This study aimed to examine the effects of LBF occurring in the late stance phase on gait performance, such as walking speed and toe-clearance, in the swing phase `and investigate the kinetic and kinematic parameters that cause LBF in stroke patients.

## Methods

### Participants

This cohort study comprised stroke patients who underwent gait analysis using a motion analysis system at Seiai Rehabilitation Hospital (Fukuoka, Japan) between 2017 and 2020.

The inclusion criteria were sub-acute stroke patients admitted to the rehabilitation unit, age > 20 years, stroke in the supratentorial area, paresis in one lower limb, ability to understand instructions for performing the gait analysis, and ability to walk 10 m without the assistance of another person or walking aids. The exclusion criteria were patients with bilateral stroke and a history of other neurological or musculoskeletal disorders unrelated to stroke; patients with the PF required to identify the LBF interval was not observed. This research was reviewed and approved by the institutional review board of Seiai Rehabilitation Hospital (approval number: 20–244). All participants gave informed consent prior to participation in the study. This study was conducted in accordance with the Declaration of Helsinki guidelines.

### Patient demographics and clinical characteristics

Age, sex, height, weight, time post stroke, stroke type (hemorrhagic or ischemic), paretic side, Fugl-Meyer Assessment (FMA) total score, FMA lower extremity motor score, FMA balance score, trunk control test (TCT) values, and functional accommodation categories (FAC) were investigated at the time of gait measurement.

#### Fugl-Meyer assessment

The FMA is a 226-point multi-item Likert-type scale developed as an evaluative measure of recovery from hemiplegic stroke. It is divided into 6 domains: upper extremity motor, lower extremity motor, sensory function, balance, joint range of motion, and joint pain. Each domain contains multiple items each scored on a 3-point ordinal scale (0 = cannot perform, 1 = performs partially, 2 = performs fully)^26,27^. The FMA lower extremity motor score is a subscale measuring lower limb motor recovery. It examines movement, coordination, and reflex action of the hip, knee, and ankle in the supine, sitting, and standing positions. The score range is 0 to 34, with higher scores indicating better lower limb motor performance^27^. The FMA balance score is a subscale measuring postural control in sitting and standing positions. It measures postural response and retention ability in the sitting, standing, and one-legged standing positions. The score range is 0 to 14, with higher scores indicating better balance ability performance^27^.

#### Trunk control test

The TCT has been developed to measure trunk control in stroke patients^28^. The TCT, besides investigating the maintenance of sitting position, further examines the ability to roll from a supine position towards the paretic side and non-paretic side sides, and supine to sitting position transfer. The score range is 0 to 100, with higher scores indicating better trunk control performance.

#### Functional accommodation categories

The FAC is an assessment of walking independence^29^. It has six levels (0 to 5) that are classified according to the walking ability based on the amount of physical support required as follows: nonfunctional ambulatory (FAC 0), continuous manual contact to support the body weight and maintain balance or to assist with coordination (FAC 1), intermittent or continuous light touch to assist with balance or coordination (FAC 2), ambulatory, dependent on supervision (FAC 3), ambulatory, independent, level surface only (FAC 4), and ambulatory, independent (FAC 5).

### Experimental conditions

Participants' task consisted of walking along an 8-m walkway at a self-selected gait speed. The gait cycle of the paretic lower limb was measured at least five times. All participants walked without using any walking aid, such as a cane or orthosis. A therapist accompanied them during gait measurement to prevent falls.

### Experimental setup

Fourteen VICON-MX cameras (Vicon Motion System Ltd., Oxford, UK) and six force plates (600 mm × 400 mm; Advanced Mechanical Technology Inc., Watertown, MA, USA) were used for data collection. The sampling frequencies of the cameras and force plates were 100 Hz and 1000 Hz, respectively. Of the 8 m experimental walking path, the measurement section was 6 m and the run-up section was 2 m. Referring to the Helen Hayes Marker Set, 29 reflective markers were attached to the participants' bodies, including one at the center of the sacrum as an additional marker for the pelvis.

### Data collection and analysis

Visual3D analytical software (C-Motion Ltd., Rochelle, IL, USA) was used for data processing. Spatial coordinates of reflective markers and GRF data were processed using low-pass filters of 6 Hz and 18 Hz, respectively. The link segment model consisted of 13 segments (head, trunk, pelvis, upper arms, forearms, thighs, shanks, and feet). The tri-axial joint angle and angular velocity of the lower limbs, the joint moment of the lower limbs, GRF in one walking cycle, and COP were calculated; the data of the joint moment and GRF were normalized using the participants' body weight.

Based on the vertical component of GRF, one gait cycle was divided into a loading response phase, a single leg support phase, a pre-swing phase, and a swing phase. The threshold of the vertical GRF was set at 1% of the body weight as per a previous study^12^.

**Figure 1:**
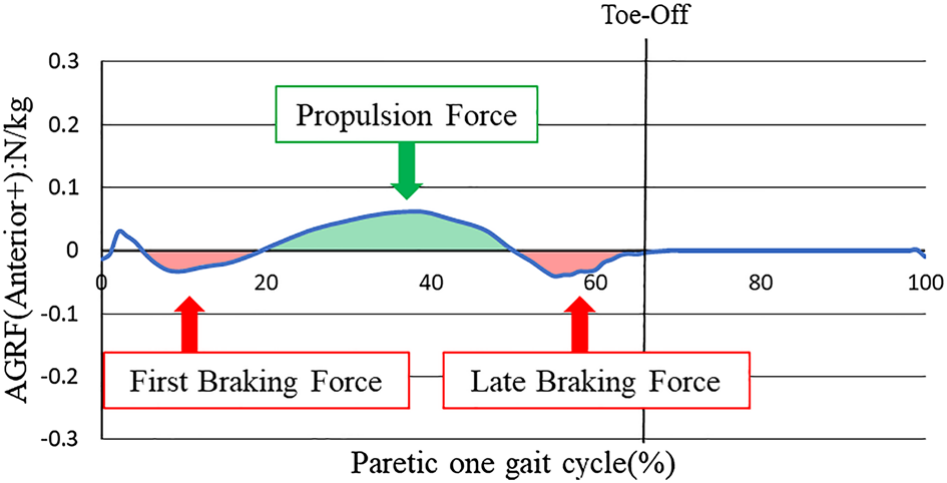
AGRF of typical cases in which LBF appears. LBF was discriminated based on the braking force occurring from PF to toe-off in the late stance phase of the paretic lower limb. AGRF: Anterior and Posterior ground reaction force.

LBF was measured in the posterior component of GRF of the paretic lower limbs after normalizing the gait cycle of the paretic lower limbs as 100%. According to a previous study^12^, the posterior component of GRF that occurred immediately after the initial contact was defined as FBF, the anterior component that occurred after FBF was defined as PF, and the posterior component that occurred after PF was defined as LBF (Fig. 1). After evaluating LBF, PF impulse and LBF impulse, which are the time integrals of PF and LBF, respectively, were calculated as per previous studies^15,30^.

The following parameters associated with LBF were selected as analytical variables: TLA, CRP-RMS, the peak value of the ankle plantar flexion moment in the pre-swing phase, and COP forward displacement distance in the paretic stance phase. COP forward displacement distance was normalized by the distance between the heel marker and the second metatarsal head marker on the sagittal plane. The ankle plantar flexion moment was normalized by the participant's weight. TLA was evaluated as the angle between the perpendicular of the floor from the greater trochanter and the line from the greater trochanter to the distal end of the hind limb. Lewek et al.^31^ reported that TLA calculated using COP as the distal end had the most similarity to PF. Hence, COP was adopted at the distal end, and the peak value of the TLA of the paretic limb in the single leg support phase was calculated.

CRP-RMS was calculated as per previous studies^32,33^. First, normalization was performed on the angles and angular velocities in the sagittal plane of the segment using the maximum value of the interval.

$$Angle\!\!:{\theta }_{\begin{array}{c}Ni\end{array}}=\frac{2\times {{[\theta }_{i}-(\theta }_{MAX}+{\theta }_{Min})/2]}{{{\theta }_{MAX}-\theta }_{MIN}}$$

$$Angle Velocity\!\!:{\omega }_{Ni}=\frac{{\omega }_{i}}{{MAX\{MAX(\omega }_{i}), MAX{(-\omega }_{i})\}}$$

The phase angle was then calculated for each segment using inverse trigonometric functions on the normalized angles and angular velocities.

$$Phase\; angle\!\!:{\varphi }_{Ni}={\mathrm{tan}}^{-1}(\frac{{\omega }_{Ni}}{{\theta }_{Ni}})$$

The difference between the phase angles was then calculated as the phase difference, and the RMS during the pre-swing phase was calculated as CRP-RMS.

$${CRP}_{segmentA\_segmentB}={\varphi }_{segmentA}-{\varphi }_{SegmentB}$$

$${CRP}_{\begin{array}{c}segmentA-SegmentB\end{array}}RMS=\sqrt{\frac{1}{N}} \sum_{i}^{N}{({CRP}_{i} )}^{2}$$

Herein, the CRP-RMS values between the pelvic and paretic thigh (CRP_Pelvis-P_Thigh_RMS), the paretic thigh and paretic shank (CRP_P_Thigh-P_Shank_RMS), and paretic shank and paretic foot (CRP_P_Shank-P_Foot_RMS) were calculated as limb coordination of paretic lower limbs. In addition, CRP-RMS values between the paretic and non-paretic thighs (CRP_P_Thigh-NP_Thigh_RMS), the paretic and non-paretic shank (CRP_P_Shank-NP_Shank_RMS), and the paretic foot and non-paretic foot (CRP_P_Foot-NP_Foot_RMS) were calculated as indicators of cooperativity with the non-paretic side. The following spatiotemporal gait parameters were calculated: walking speed, step length on the paretic limb, step length on the non-paretic limb, loading response phase time of the paretic leg, single leg support phase time, pre-swing phase time, swing phase time, the peak value of the paretic knee flexion angles in the pre-swing and swing phases, and toe clearance during the paretic swing phase. Toe clearance was defined as the distance of the perpendicular line between the head of the second metatarsal bone marker and the floor at the time when the paretic and nonparetic ankle joint markers crossed the sagittal plane during the paretic swing phase. The step length and tip-to-floor distance were normalized to the participants' height.

### Statistical analysis

Statistical analyses were performed using SPSS Statistics Ver.28 (International Business Machines Corp., Armonk, NY, USA). The mean value of the five gait cycles of the paretic limb was used for all gait analysis data. The normality of each parameter was confirmed using the Shapiro–Wilk test; the significance level was set at *p* < 0.05. First, comparisons of spatiotemporal and kinematic/kinetic parameters of gait in stroke patients with and without LBF were analyzed by either an independent-samples t-test or Mann–Whitney's U test. Second, regarding the effect of LBF on gait performance, since PF impulse has been reported to affect the spatiotemporal parameters of gait^8,9^, we calculated partial correlation coefficients between LBF impulse and spatiotemporal parameters with PF impulse as a control variable to remove this effect. In addition, the linear relationships between LBF impulse and the kinetic and kinematic parameters, TLA, peak paretic ankle plantar flexion moment, COP forward displacement distance, and each CRP-RMS, were evaluated using Pearson's correlation coefficient. Finally, stepwise multiple linear regression analysis was performed with LBF impulse as a response variable and kinetic and kinematic parameters as explanatory variables to identify kinetic and kinematic parameters independently associated with LBF impulse and investigate their relative contributions.

## Results

A total of 235 patients with stroke were hospitalized during the study inclusion period. Of these patients, 148 were excluded from the study, and 85 met the inclusion criteria and were enrolled in the study. Basic demographic and clinical characteristics are shown in Table 1. Of the 157 stroke patients in this case group, 110 had LBF in the late paralytic side stance phase.

**Table 1:** Basic demographic and clinical characteristics.

Variable	Value
Age (years) [mean ± SD]	61.8 ± 12.0
Sex (n) [female/male]	102 / 55
Height (m) [mean ± SD]	1.63 ± 0.08
Weight (kg) [mean ± SD]	59.8 ± 10.8
Time post-stroke (days) [mean ± SD]	100.3 ± 50.7
Type of stroke (n) [hemorrhagic/ischemic]	70 / 87
Paretic side (n) [left/right]	74 / 83
FMA total score [mean ± SD]	184.0 ± 29.4
FMA lower extremity motor score [mean ± SD]	27.2 ± 5.6
FMA balance score [mean ± SD]	10.6 ± 2.0
Trunk control test [mean ± SD]	92.0 ± 13.3
FAC (n) [III/IV/V]	58/62/37

The number of patients: 157.

*FMA* Fugle Meyer assessment, *FAC* functional ambulation categories, *COP* center of pressure, *CRP* continuous relative phase, *P* paretic side, *NP* non-paretic side, *RMS* root mean square, *LBF* late braking force, *PF* propulsion force, *SD* standard deviation.

### Comparison of spatiotemporal and kinematic and kinetic parameters of gait with and without LBF

A comparison of the spatiotemporal and kinematic and kinetic parameters of gait with and without LBF is shown in Table 2. Patients with LBF showed significant reductions in most spatiotemporal parameters and kinematic and kinetic parameters; however, COP Forward Displacement Distance, CRP_P_Shank-NP_Shank_RMS, CRP_P_Foot-NP_Foot_RMS were not significantly different.

**Table 2:** A comparison of the spatiotemporal and kinematic and kinetic parameters of gait with and without LBF.

Variable	LBF − (n = 47) mean ± SD	LBF + (n = 110) mean ± SD	*p *value
Spatiotemporal parameters			
Walking speed (m/sec)	0.80 ± 0.22	0.56 ± 0.26	< 0.001
Paretic step length (m/height)	0.28 ± 0.05	0.23 ± 0.07	< 0.001
Non-paretic step length (m/height)	0.28 ± 0.05	0.22 ± 0.07	< 0.001
LR time (sec)	0.16 ± 0.05	0.29 ± 0.26	< 0.001
SS time (sec)	0.41 ± 0.05	0.39 ± 0.08	0.035
PSw time (sec)	0.16 ± 0.04	0.29 ± 0.22	< 0.001
SW time (sec)	0.44 ± 0.06	0.55 ± 0.16	< 0.001
PSw_paretic knee flexion peak (deg)	39.4 ± 7.0	32.6 ± 11.0	< 0.001
SW_paretic knee flexion peak (deg)	55.1 ± 8.1	39.5 ± 13.5	< 0.001
Toe clearance (m/height)	0.04 ± 0.01	0.04 ± 0.01	0.042
Kinematic and kinetic parameters			
PF impulse (N/kg*s)	1.55 ± 0.70	0.78 ± 0.64	< 0.001
Tailing limb angle (deg)	12.7 ± 3.5	8.1 ± 4.7	< 0.001
Ankle plantar flexion moment peak (Nm/Kg)	0.93 ± 0.28	0.76 ± 0.26	< 0.001
COP forward displacement distance (m/Foot length)	0.80 ± 0.23	0.73 ± 0.28	0.144
CRP_Pelvis-P_Thigh_RMS (deg)	61.9 ± 38.9	76.9 ± 43.2	0.032
CRP_P_Thigh-P_Shank_RMS (deg)	143.6 ± 6.7	131.3 ± 23.9	< 0.001
CRP_P_Shank-P_Foot_RMS (deg)	11.8 ± 4.7	15.8 ± 7.9	< 0.001
CRP_P_Thigh-NP_Thigh_RMS (deg)	163.7 ± 9.2	147.6 ± 18.3	< 0.001
CRP_P_Shank-NP_Shank_RMS (deg)	28.8 ± 25.8	37.0 ± 22.2	0.063
CRP_P_Foot-NP_Foot_RMS (deg)	73.2 ± 21.6	84.9 ± 29.9	0.144

*LBF* + LBF appears, *LBF − *LBF does not appear, *LR* loading response phase, *SS* single leg support phase, *PSw* pre-swing phase, *SW* swing phase, *PF* propulsion force, *COP* center of pressure, *CRP* continuous relative phase, *P* paretic side, *NP* non-paretic side, *RMS* root mean square, *SD* standard deviation.

### Relationship between LBF impulse and spatiotemporal parameters with PF impulse as a control

The relationships between LBF impulse and spatiotemporal parameters with PF impulse as a control variable are shown in Table 3. Weak significant correlations were found for walking speed, step length on the non-paretic limb, loading response phase time, pre-swing phase time, swing phase time, and toe clearance. Furthermore, the peak value of the paretic knee flexion angle in the pre-swing phase and swing phase showed moderately significant correlations, but the step length on the paretic limb and single leg support phase time did not show significant correlations.

**Table 3:** Relationship between LBF impulse and spatiotemporal parameters with PF impulse as a control.

Variable	Correlation coefficient	*p* value
Walking speed (m/sec)	− 0.26	< 0.001
Paretic step length (m/height)	− 0.14	0.08
Non-paretic step length (m/height)	− 0.27	< 0.001
LR time (sec)	0.17	0.034
SS time (sec)	− 0.15	0.056
PSw time (sec)	0.27	< 0.001
SW time (sec)	0.33	< 0.001
PSw_paretic knee flexion peak (deg)	− 0.40	< 0.001
SW_paretic knee flexion peak (deg)	− 0.48	< 0.001
Toe clearance (m/height)	− 0.27	< 0.001
FAC	0.022	0.781

The number of patients: 157.

*LR* loading response phase, *SS* single leg support phase, *PSw* pre-swing phase, *SW* swing phase, *FAC* functional ambulation categories, *LBF* late braking force, *PF* propulsion force.

### Linear relationship between LBF impulse and kinetic and kinematic parameters

The linear relationships between LBF impulse and kinetic and kinematic parameters are shown in Table 4. COP Forward Displacement Distance and CRP_P_Shank-NP_Shank_RMS and CRP_P_Foot-NP_Foot_RMS showed a weak significant correlation. Furthermore, TLA, Ankle Plantar Flexion Moment Peak, CRP_P_Thigh-P_Shank_RMS, and CRP_P_Thigh-P_Shank_RMS showed moderately significant correlations, and CRP_P_Thigh-NP_Thigh_RMS showed a strong correlation. In contrast, CRP_Pelvis-P_Thigh_RMS showed no significant correlation.

**Table 4:** The linear relationship between LBF impulse and the kinetic and kinematic parameters.

Variable	Correlation coefficient	*p* value
Trailing limb angle (deg)	− 0.60	< 0.001
Ankle plantar flexion moment peak (Nm/Kg)	− 0.48	< 0.001
COP forward displacement distance (m/Foot length)	− 0.23	0.004
CRP_Pelvis-P_Thigh_RMS	0.13	0.274
CRP_P_Thigh-P_Shank_RMS	− 0.47	< 0.000
CRP_P_Shank-P_Foot_RMS	0.54	< 0.001
CRP_P_Thigh-NP_Thigh_RMS	− 0.78	< 0.001
CRP_P_Shank-NP_Shank_RMS	0.31	< 0.001
CRP_P_Foot-NP_Foot_RMS	0.28	0.002

The number of patients: 157.

*COP* center of pressure, *CRP* continuous relative phase, *P* paretic side, *NP* non-paretic side, *RMS* root mean square, *LBF* late braking force, *PF* propulsion force.

### Multiple regression analysis using kinetic and kinematic parameters and their relative contributions to LBF impulse

The results of the multiple linear regression analysis are shown in Table 5. Multiple linear regression analysis showed that the main determinants of LBF impulse were TLA (β = − 0.174, *p* = 0.005), CRP_P_Shank-P_Foot_RMS (β = 0.147, *p* = 0.011), and CRP_P_Thigh-NP_Thigh_RMS (β = − 0.599, *p* < 0.001). The coefficient of determination with adjusted degrees of freedom was 0.644, and the variance inflation factor ranged from 1.7 to 1.8, indicating the absence of multicollinearity. Created model equations were significant (*p* < 0.001).

**Table 5:** Kinetic and kinematic parameters independently related to LBF impulse and relative contributions.

	Unstandardized coefficients	Standard error	β	*p* value	95% CI		VIF
					Lower limit	Upper limit	
Intercept	0.524	0.051		0.000	0.340	0.661	
Trailing limb angle	− 0.003	0.001	− 0.174	0.005	− 0.007	0.000	1.595
CRP_P_Shank-P_Foot_RMS	0.002	0.001	0.147	0.011	0.004	0.008	1.426
CRP_P_Thigh-NP_Thigh_RMS	− 0.003	0.000	− 0.599	0.001	− 0.004	− 0.002	1.752

The number of patients: 157 R^2^ = 0.650 Adjusted R^2^ = 0.644.

*LBF* late breaking force, *β* standardized coefficients, *VIF* variance inflation factor, *CI* confidence interval, *CRP* continuous relative phase, *RMS* root mean square.

## Discussion

Some studies have investigated the relationship between the sum of propulsive and braking forces and lower limb muscle activity occurring in the late stance phase of the paretic lower limb in stroke patients^15^. Others have demonstrated that LBF is associated with paretic lower limb kinematics during the pre-swing phase^12^; however, the role of LBF has not been thoroughly verified.

This is the first study to show the effect of LBF on gait performance and examine the kinetic and kinematic parameters that contribute to the development of LBF in stroke patients. Herein, of the 157 stroke patients, 110 exhibited LBF in the late stance phase of the paretic limb. This suggests that LBF appears in the gait of most stroke patients. Accordingly, it is vital to examine the association and effects of LBF to improve the gait function of stroke patients. Furthermore, patients with LBF had lower spatiotemporal parameters of gait than those without LBF, suggesting that the should be more focus on LBF as a component of gait disturbance in stroke patients.

Analysis of the relationship between LBF impulse and spatiotemporal parameters with PF impulse as the control variable indicated a negative correlation between LBF and walking speeds, non-paretic step lengths, toe clearances, and knee joint flexion angles during the pre-swing and swing phases, and a positive correlation between LBF and loading response time, pre-swing phase time, and swing phase time.

However, most variables had low correlation coefficients, and only knee joint flexion angles in the pre-swing and swing phases showed moderate correlations. This result is similar to that reported by Dean et al.^12^, suggesting that not only a decrease in PF but also an increase in LBF decreases knee joint flexion angles during the pre-swing and swing phases. This is thought to be because the mechanical energy of push-off due to PF is reduced by LBF that occurs just before toe-off, thus decreasing the knee joint flexion angle. Therefore, for the decrease in knee joint flexion angles during the pre-swing and swing phases, attention must be paid not only to promoting an increase in PF but also to a decrease in LBF.

Since the knee flexion angles during the pre-swing and the swing phases were negatively correlated with LBF impulse, we expected a significant correlation between LBF impulse and toe clearance; however, contrary to our prediction, the correlation coefficient between the LBF impulse and toe clearance was low. This is because toe clearance is compensated by pelvic elevation when functional shortening of the paretic lower limb is reduced during the swing phase, which has been shown by previous studies^34,35^, and we speculate that similar compensatory movements may be involved herein.

Turns et al.^15^ reported that braking force occurred in the late stance phase of the paretic lower limb in patients with low lower limb function and slow walking speed. However, we observed a weak correlation between LBF impulse and walking speed. This may be because we performed a partial correlation analysis using PF impulse as a control variable, and it has been reported previously that PF is strongly involved in walking speed^8,9^. Furthermore, LBF occurring immediately before toe-off only slightly inhibits the forward propulsion of the body.

FAC, which indicates walking independence, showed no relationship with LBF. In stroke patients, FAC has been reported to be related to walking speed^36^, balance ability^37^, cognitive function^38^, and a variety of factors. Therefore, it is unlikely that LBF has a direct influence on FAC. These results suggest that LBF does not significantly affect toe clearance, walking speed, or walking independence, but is a factor that decreases knee joint flexion angle in the pre-swing and swing phases independent of PF.

Significant relationships between LBF impulse and kinetic and kinematic parameters, except CRP_Pelvis-P_Thigh_RMS, were found. Furthermore, multiple regression analysis showed that CRP_P_Thigh-NP_Thigh_RMS, CRP_P_Shank-P_Foot_RMS, and TLA were factors that affected LBF impulse.

A CRP of 0° indicates that the segments move in-phase; as the CRP increases, the two segments are increasingly out-of-phase until a CRP of 180° that indicates reverse-phase coupling is seen^32,33^. Our results showed a negative correlation between LBF impulse and CRP_P_Thigh-NP_Thigh_RMS, which can be interpreted as a tendency for LBF to increase when the motion of the paretic and non-paretic thighs is near in-phase, and for LBF to decrease when they are near reverse-phase during the paretic pre-swing phase. A previous study reported that the magnitude of flexion motion and the angular velocity of flexion were markedly lower in the paretic hip than in the non-paretic hip in the paretic pre-swing phase and initial swing phase^39^. It is suggested that stroke patients are more likely to exhibit LBF due to decreased interlimb coordination between the paretic and non-paretic hip during the pre-swing phase.

Second, a negative correlation was observed between LBF impulse and TLA. Since the magnitude of TLA is directly related to PF^20–22^, this suggests that stroke patients with smaller PF have larger LBF, as reported in a previous study^15^. Moreover, TLA is related to the knee joint flexion angle during the pre-swing phase^40^. Hence, a decrease in TLA might have delayed the de-loading of the paretic limb during the pre-swing phase and contributed to the development of LBF.

The positive correlation between LBF impulse and CRP_P_Shank-P_Foot_RMS can be interpreted as a tendency for LBF to increase when the paretic limb and foot are close to reverse-phase motion and for LBF to decrease when the paretic limb and foot are close to in-phase motion during the paretic pre-swing phase. It has been reported that immobilization of the ankle and metatarsophalangeal joint inhibits forefoot rocker and toe rocker, obtained by foot and lower leg coordination, and significantly reduces PF and ankle joint push-off in the late stance phase^41,42^. The results of this study indicate that loss of coordinated movement of the paretic foot and shank increases the likelihood of LBF.

This study has several limitations. First, we limited the parameters to be analyzed based on our prior hypotheses and did not conduct an exploratory validation. Therefore, we cannot deny the possibility that there are factors other than the parameters used in this study related to the occurrence of LBF. Second, it has been reported that limb kinematics is more valuable than joint kinematics in explaining gait performance^43^; therefore, it is necessary to investigate limb kinematic parameters related to LBF. Third, LBF may not be specific to stroke patients; therefore, geriatric populations and patients with gait disturbance due to other diseases should be investigated and compared. Finally, the present study did not provide recommendations for methods to improve LBF, which is predicted to benefit from methods to improve PF due to the similar kinematic and kinetic properties associated with PF. Specifically, training to improve PF has included fast gait training^44^, Fast-FES training^45^, and real-time biofeedback gait training using anterior–posterior GRF on the paretic limb^46^. However, these methods have not been verified to suggest an improvement in LBF, and future verification is needed.

In conclusion, although LBF occurring during the late stance on the paretic limb does not directly affect toe-clearance or gait independence, it negatively affects spatiotemporal gait parameters, mainly during the pre-swing and swing phases on the paretic limb, and thus should be given more attention to improve gait impairment in stroke patients. In addition to TLA in the late stance of the paretic limb, which is strongly associated with PF, the limb coordination of the foot, shank, and both hip joints in the pre-swing phase of the paretic limb may be significantly involved in the development of LBF.

## Data Availability

The datasets generated during and analysis during the current study are available from the corresponding author on reasonable request.
